# Lentivirus-mediated RNA interference of clusterin enhances the chemosensitivity of EJ bladder cancer cells to epirubicin *in vitro*

**DOI:** 10.3892/mmr.2021.12267

**Published:** 2021-07-01

**Authors:** Jian Lu, Jun-Hang Luo, Jian Pang, Jia-Zheng Cao, Rong-Hai Wu, Zhu-Ting Tong, Wei Chen, Dan Xie

Mol Med Rep 6: 1133-1139, 2012; DOI: 10.3892/mmr.2012.1017

Subsequently to the publication of the above paper, the authors have realized that Fig. 2A in this paper contained an error. The image selected to represent the experiment showing the invasion ability of EJ cells in the epirubicine/LV-NC group of Fig. 2A was chosen mistakenly during the figure compilation process.

A corrected version of Fig. 2 is shown on the next page. Note that this error did not affect either the results or the conclusions reported in this paper, and all the authors agree to this Corrigendum. The authors are grateful to the Editor of *Molecular Medicine Reports* for allowing them the opportunity to publish this Corrigendum, and apologize to the readership for any inconvenience caused.

## Figures and Tables

**Figure 2.**
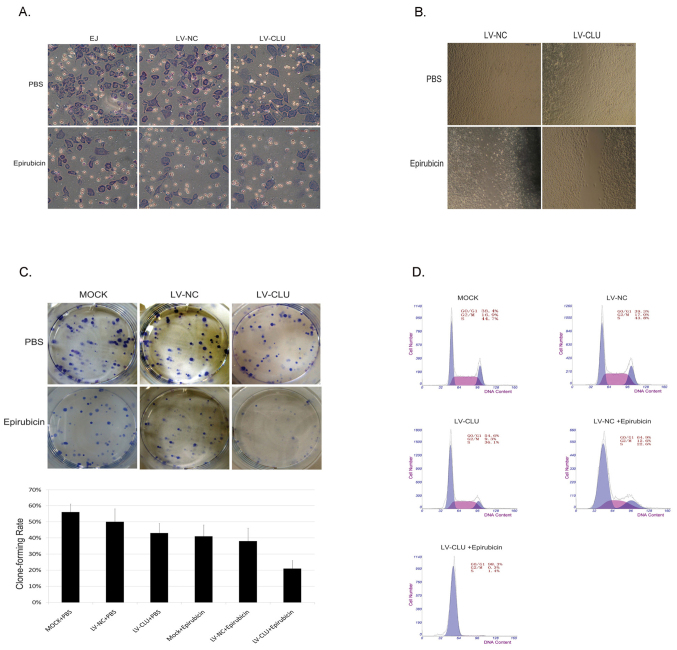
Analysis of the role of CLU in chemotherapeutic resistance. (A) A Matrigel invasive assay demonstrated that LV-CLU-infected cells combined with epirubicin treatment had minimum invasiveness. Statistical analysis revealed a significant difference between the LV-NC and LV-CLU groups with epirubicin treatment (P<0.01) (n=5). (B) A wound healing assay was used to detect the migration ability of EJ cells after CLU knockdown. LV-NC- and LV-CLU-infected EJ cells were treated with or without epirubicin, respectively, for 22 h. LV-CLU-infected EJ cells had a greater migrating distance compared to LV-NC infected EJ cells which were almost confluent. A significant difference was observed between the two groups (P<0.05) (n=6). (C) A plate clone formation assay with or without epirubicin treatment is shown. The data demonstrated that CLU knockdown EJ cells combined with epirubicin treatment had a lower clone formation rate than the other groups (P<0.05) (n=3). (D) Effect of CLU knockdown on the cell cycle detected by flow cytometric analysis. CLU-silenced cells demonstrated G0/G1 phase arrest and G2/M and S-phase reduction. After treatment with epirubicin for 24 h, 98.3% cells were blocked in the G0/G1 phase in CLU knockdown EJ cells, while only 64.9% cells were blocked in the G0/G1 phase in LV-NC-infected cells. The difference was statistically significant. CLU, clusterin; LV-CLU lentivirus targeting CLU; LV-NC, lentivirus targeting negative control.

